# Nurses’ perspectives on clinical competence to identify loneliness and depression in older people in home care: a qualitative study

**DOI:** 10.1186/s12913-025-12428-y

**Published:** 2025-03-12

**Authors:** Birgit Hauger, Randi Martinsen, Knut Hestad, Liv Skomakerstuen Ødbehr

**Affiliations:** 1 Inland Norway University of Applied Sciences, Elverum, Norway; 2 Inland Hospital, Hamar, Norway

**Keywords:** Nursing competence, Home care, Qualitative approach, Clinical nursing knowledge, Older people, Home-dwelling, Person-centred care

## Abstract

**Background:**

Loneliness and depression in older people are increasing worldwide. Proficient nursing practice requires the continuous and long-term development of clinical knowledge. Lifelong learning is essential and enhances clinical nursing practice. Nursing includes various functional areas that illustrate registered nurses’ wide range of responsibilities to ensure healthcare quality. Registered nurses are committed to working comprehensively, observing patients and identifying symptoms of both physical and mental ill-being. The aim of this study is to explore how registered nurses perceive their clinical competence in observing signs of loneliness and depression in older people in home care, and which barriers in home care services prevent quality improvement.

**Methods:**

Fifteen registered nurses from home care units in Norway were individually interviewed, and the interviews were analysed using qualitative content analysis.

**Results:**

The analysis revealed three themes in accordance with the study aim: I) Uncertainty about knowledge of mental health, II) Lack of opportunities to meet the patients’ needs regarding mental ill-being, III) Feelings of guilt for not doing enough for patients.

**Conclusions:**

Lack of education and training in mental health creates uncertainty and limits registered nurses’ ability to effectively address signs of loneliness and depression. Registered nurses often feel unprepared to handle psychological aspects of care.

Organizational barriers hinder effective care. Additionally, the emotional toll on registered nurses, who experience guilt and moral distress due to their limited ability to provide sufficient care, highlights the impact of these systemic issues on patients.

The study underscores the need for improved training, better collaboration, and stronger leadership to prioritize mental health and ensure that registered nurses have the necessary resources and support to meet the needs of older people with signs of mental ill-being in home care.

**Supplementary Information:**

The online version contains supplementary material available at 10.1186/s12913-025-12428-y.

## Introduction

### Increased prevalence of mental ill-being worldwide

Increased longevity is a worldwide phenomenon [1], and many older people are receiving healthcare at home. In Norway, average life expectancy is projected to rise from the current figures of 80 years for men and 84 years for women to 87 and 89 years, respectively, in the year 2060. Consequently, the population will contain a greater proportion of older people [2]. Between 30 and 50% of adults experience signs of mental ill-being at some point in their lives [3]. The World Health Organization emphasizes that mental health disorders are frequent [4] and that signs of loneliness and depression among older people are on the rise globally [5, 6]. Loneliness has now emerged as a significant public health and societal challenge [7], and there is a clear connection between loneliness and negative health consequences [8–10]. This issue has become particularly pronounced in recent years among older people, especially those living alone [11]. Although loneliness is not classified as a mental disorder, the consequences of loneliness can be depression and other types of mental ill-being or conditions [10], such as poor self-rated health, limited physical abilities and multi-morbidity [8]. The impact of loneliness therefore represents a significant public health issue [7], and individuals who displayed a higher level of loneliness had greater anxiety, depressive symptoms and irritability [12]. A focus on loneliness from a preventative perspective is considered an important objective in primary healthcare [10]. Signs of mental ill-being in general increases with age and differs between varied groups of older people [9, 13–15]. The incidence of depression increases most in the age group over 75 years, especially in those who have physical illnesses [16]. Research shows that one in five older people experience depressive symptoms, and two out of three experience a moderate sense of loneliness [12]. Signs of mental ill-being in older people can be mistaken for dementia or just attributed to old age. This may lead to symptoms of loneliness and depression being undiscovered and underdiagnosed in this group [16–22]. Many older people find it difficult to talk about their mental health [15], and further, this makes it difficult for home care nurses to understand their feelings [23]. Research shows that the higher the quality of nursing education in elderly care, the lower the level of loneliness [24]. Home care nurses’ close contact with patients puts them in a unique position to observe their mental health, including changes in mental health [16]. Moreover, nurses in home care settings find it difficult to meet older peoples’ needs [25], which is primarily due to organizational structures. Yet they have to face this challenge in their daily communication with patients [23]. However, research has shown that home care nurses were more concerned about practical home care tasks than detecting signs of mental ill-being and promoting mental health [25].

### Aim

The study aim is to explore registered nurses’ perception of their clinical competence to identify signs of loneliness and depression in older people in home care, and barriers in healthcare services that prevent quality improvement.

### Person-centred care

Person-centred care is described as a method of practice based on building and nurturing therapeutic relationships among care providers, patients, and those significant in the patients' lives. This approach is grounded in principles of respect for individuals, the right of self-determination, mutual understanding, and shared respect. It is supported by empowering cultures that promote ongoing development in practice [26]. A person-centred approach empowers patients by giving them the knowledge and confidence to participate actively in their care [27]. The Health Foundation [27] states that person-centred care is grounded in treating people with dignity, compassion and respect, ensuring that care and support are coordinated and tailored, and helping individuals recognize and build on their strengths to lead independent and fulfilling lives. Enabling person-centred care requires a partnership between registered nurses and patients, where they work together to understand the patients’ needs, make decisions and set goals. Person-centred care focuses on the whole person, beyond their condition. In addition to its ethical foundation, person-centred care brings significant practical benefits by enhancing patient experiences, improving the quality of care, and positively impacting health outcomes. This approach helps patients manage their health more effectively, which reduces emergency visits and fosters better adherence to treatment. When patients are actively engaged in decision-making, they report greater satisfaction with their care, and treatments tend to be less costly. Furthermore, person-centred care supports registered nurses by boosting their morale and performance, ultimately delivering better value by closely aligning care with the specific needs of each patient [27]. Actively involving patients and placing them at the centre of their care is now recognized as essential to providing high-quality healthcare [28]. Person-centred care emphasizes recognizing older home-dwelling people as unique individuals and engaging them as active partners in their care [27]. In the context of home care, this approach is particularly important, as nurses working with older people must tailor care to meet the specific needs of each person in their living environment. By ensuring coordinated and personalized care efforts, home care nurses can empower older people to actively participate in their health management. This collaborative approach not only enhances the patient’s overall experience but also fosters self-esteem by promoting positive self-perception, even in old age, and supports self-efficacy by building confidence in the person’s ability to manage personal health goals [27, 29, 30]. The relationship between the registered nurse and the patient plays a crucial role, as the nurse’s consistent and compassionate engagement is central to the patient’s sense of dignity and well-being [27].

### The role of municipal healthcare and nursing role

The Municipal Health Services Act ensures that municipalities provide their residents with access to necessary healthcare services. In Norwegian municipalities, various healthcare services cater to different needs. Mental health services provide follow-up care for those facing mental health challenges and disorders while home care delivers comprehensive health services that involve medical treatment, nursing care and support services provided in a patient's home by healthcare professionals. It allows individuals to receive necessary treatment and assistance with daily activities while staying in a familiar environment. Home care delivers comprehensive health services that involve medical treatment, nursing care and support services provided in patients home by healthcare professionals. It allows individuals to be supported with daily activities while stay in a familiar environment. Home care is essential for managing chronic conditions and post-hospital care, improving patient outcomes, and promoting independence. Home care nursing plays a crucial role in promoting health, preventing disease, and managing chronic illnesses in various global healthcare systems. This form of care is aimed to enhance patients’ outcomes by ensuring continuity of treatment and support the patients’ emotional and psychological well-being by allowing them to maintain a sense of independence and familiarity within their homes. Thus, home care is a multifaceted and flexible concept that aims to enable individuals to remain in their home environment while receiving necessary support. It integrates a wide range of services tailored to individual needs, blending both social and health care provided through a professional caregiving [31].

Registered nurses are healthcare professionals who have completed the necessary education and licensing requirements to practice nursing. In Norway, students must complete a three-year university program to reach a bachelor's degree in nursing. Registered nurses have various functional areas that demonstrate the breadth of nursing responsibilities for providing high-quality healthcare [32]. Registered nurses are among the professional groups working within municipal healthcare services, collaborating closely with other professionals such as healthcare workers, social educators, and social workers. Their tasks are guided by decisions regarding assistance needs, made by the municipality's allocation team, and their work is regulated by relevant laws and frameworks governing their roles. Registered nurses are obligated to work comprehensively [33], which implies observing and identifying signs of illness in both physical and mental health. Research shows that nurses are motivated to enhance their knowledge, improve their skills, and keep up-to-date with recent evidence [34]. Registered nurses’ education, experience, abilities, attitudes and values are all important factors in the delivery of home care and influence how assessments, clinical judgements and decisions are made [33, 35, 36].

### Norwegian legislation

The public administration act regulates how public authorities handle cases, emphasizing legal safeguards, transparency, the right to appeal, and impartiality [37]. The health personnel act sets requirements for the professional conduct of healthcare personnel, including confidentiality, sound practice, and the duty to report, ensuring safe and ethical healthcare services [38]. The municipal health and care services act regulates the municipalities' responsibility to provide necessary health and care services to their residents [33]. The patients' rights act ensures patients' rights to healthcare, with a focus on information, participation, consent, and the right to appeal [39]. The personal data act regulates the processing of personal data to protect individual privacy in accordance with GDPR regulations [40]. The Working Environment Act ensures a safe and health-promoting working environment. It requires employers to prevent health and safety risks and provide necessary training [41]. Norwegian municipalities are obliged by law to offer primary healthcare to people with physical and mental health challenges [33]. In addition, many municipalities provide non-statutory services to residents based on individual healthcare needs, and patients’ rights to primary care are ensured through legislation [39]. Patients are thus entitled to municipal healthcare, and individual needs are met based on the Public Administration Act [37]. Effective nursing practice requires long-term and ongoing acquisition of clinical knowledge [42]. The registered nurse’s role in home care is governed by the Health Personnel Act [38], the Municipal Health Services Act [33], and professional ethical guidelines [43]. Continuing professional development is central to nurses’ lifelong learning, and an important element of nursing practice.

## Methods

### Design

This study has a qualitative design based on individual interviews with home care nurses from different municipalities in different areas of Norway. Individual interviews were conducted to explore the research topic and enhance knowledge and understanding of how these nurses perceive their observational skills and whether there are barriers hindering quality improvement.

### Recruitment and participants

To be included in the study, participants had to be registered nurses working in home care with at least two years of clinical experience in the field.

Invitations to participate were sent to municipal leaders and home care managers in thirty-five municipalities, explaining the aim of the project and asking them to inform home care nurses about the study for potential recruitment. Eleven participants joined the study after contacting the first author by email with a desire to participate. An additional four participants were recruited by using the snowball sampling method. These four participants were introduced to the researcher by other home care nurses, and they all met the inclusion criteria for participation. A total of fifteen home care nurses were therefore recruited for individual interviews. There were thirteen female and two male participants, aged from 24 to 66 years of age. They had between two and twenty-three years of professional nursing experience in home care. Two of the participants worked in the northern part of Norway, four participants worked in the western part of Norway, six participants worked in the eastern part of Norway, and three participants worked in the southern part of Norway. In the recruitment, participants from all regions of Norway were included, representing both urban and rural municipalities. Even though home care is organized differently across municipalities, no new information emerged.

### Data collection

The fifteen individual interviews were conducted from November 2023 to February 2024. Ten interviews were conducted in the offices of the home care services, while five were conducted by telephone due to the great distance between the participants and the researcher. An interview guide [44] covered the topics of identifying and observing signs of loneliness and depression in older people patients during home care visits. The questions in the interview guide focused on the participants' experiences with observing symptoms of loneliness and depression in patients, any potential barriers to observing these symptoms, and how they experienced/felt about working in home care (see Table 1). The interviews followed a methodological approach that allowed the participants to speak openly about their experiences with their observational skills when dealing with home-dwelling patients who might exhibit signs of loneliness and depression. The interviews lasted between thirty and forty minutes and were recorded and transcribed verbatim.

**Table 1:** The interview guide

1. Please tell me about your experience of observing loneliness and depression in patients. What do you observe, and how do you observe it? -Positive experiences? -Negative experiences?
2. Describe your experience of potential barriers of observing loneliness and depression? -What do you think about this?
3. What feelings do you have related to working in home healthcare? How does work in home healthcare influence your work as a professional nurse? -Positive or/and negative experiences?

### Analysis

The transcribed interviews were analysed using qualitative content analysis as described by Graneheim and Lundman [45]. An inductive approach was employed, focusing on patterns in similarities and differences in the data [46]. From the beginning of the manifest content analysis, the transcribed interviews were read multiple times to gain an overall impression of the content. Based on the study aim, the interviews were reread and sorted into meaning units [47]. The meaning units condensed the manifest content into codes and sub-categories while preserving the core meaning [45]. Reflection and discussion in the research team led to interpretation of the sub-categories and comparison between them to examine similarities and differences. After grouping all sub-categories based on common characteristics, they were organized into themes that represent the underlying patterns in the data. This allows for extracting depth and meaning from the qualitative material and placing it within a broader context [47]. Interpretations of the latent content were actively pursued throughout the analysis, involving the analysis of underlying meanings that were not immediately obvious [48]. See Table 2 for an example of the analysis process.

**Table 2:** Example of analysis process

Meaning units	Condensed meaning units	Codes	Sub-categories	Themes
I feel confident when I’m with patients, but I don’t feel confident in the field of mental health. I think it partly has to do with not knowing what to do with the answers I get if someone says they’re lonely or depressed	Feels confident when she is with patients Does not feel confident in mental health as a field Uncertainty about handling mental health issues	Confident with patients Insecure about mental health Uncertainty in handling	Confidence and insecurity in practice Uncertain about how to handle responses about loneliness and depression	**Uncertainty about knowledge of mental health**
I don’t have much time to talk about mental health when I’m with patients, so I end up not asking them about it. I just sort of do what I have to do. When I think about it, it’s actually a way of avoiding responsibility…	Does not have much time to talk about mental health issues with patients Avoids asking about mental health due to time pressure Only performs necessary tasks Sees her pattern of behaviour as a form of avoidance of responsibility	Lacks time for conversation Only does what is necessary Avoidance of responsibility Recognition of the importance of mental health	Insufficient time allocated for mental health discussions Avoidance of challenging or uncomfortable topics Limited opportunities to address mental health concerns Acknowledging the impact of mental health on patient well-being	**Lack of opportunities to meet the patients' needs regarding mental ill-being**
I often feel upset when I leave patients, with a gut feeling that I should have done more for them……. For some, the home care nurse might be the only visit they receive that day	Nurses often feel that they should have done more for the patients Uncomfortable feeling in the stomach after leaving patients	Feeling of inadequacy Uncomfortable feeling	Nurses are affected and feel guilty Unpleasant feeling after leaving patients Feeling inadequate	**Feelings of guilt for not doing enough for patients**

### Ethics approval and consent to participate

The Norwegian Agency for Shared Services in Education and Research (SIKT) [49] granted approval to conduct the study (No. 467880/2023). The study was submitted for approval by the ethical committee, and the Regional Committee for Medical and Health Research Ethics (REK) approved the study (No. 478516/2023).

The guidelines for risk and vulnerability analysis of the General Data Protection Regulation were followed [40]. An electronic platform at the University of Oslo was used to collect, store and analyse data. This platform enables the collection of sensitive information for the Services for Sensitive Data system (TSD), which meets the strict legal requirements for digital processing and storage of sensitive research data [50].

The study was performed in accordance with the Declaration of Helsinki [51]. The interviews were based on voluntary, written informed consent, protection against harm, anonymity and the opportunity to review and withdraw from the study. Informants received written and oral information before the study and received and signed an informed consent form before participation [44, 51, 52]. The names of the participants were replaced with numbers, and the names were kept separate from the transcripts.

## Results

The analysis revealed three themes describing how the participants perceived their clinical competence in observing signs of loneliness and depression in older people in home care, and which barriers in healthcare services prevented quality improvement: I) Uncertainty about knowledge of mental health, II) Lack of opportunities to meet the patients’ needs regarding mental ill-being, III) Feelings of guilt for not doing enough for patients.

The findings are presented below, where statements are marked with the number of the participant.

### Uncertainty about knowledge of mental health

The findings revealed that the participants experienced ambivalence between confidence in practice and uncertainty in knowledge. The participants were apprehensive about the answers patients might give them if they asked questions and afraid of inadequacy in their patient encounters. Most reported having insufficient knowledge about mental health, as one said:

“I feel confident when I’m with patients, but I don’t feel confident in the field of mental health. I think it partly has to do with not knowing what to do with the answers I get if someone says they’re lonely or depressed”. (Participant 15)

Another participant stated:

“I try my best to absorb all the knowledge I can from colleagues and the internet. However, it is so complex that it becomes challenging. This is something one needs to experience firsthand to truly learn, not just read about”. It became evident that experience was desired to build confidence”. (Participant 1)

The results showed the importance of relationships and highlighted that home care nurses are in an ideal position to foster good relationships with patients. Several participants frequently visited patients and had a unique opportunity to build relationships, as one of them stated:

“In home care, we can build a relationship with a patient. We spend a lot of time with the patients and get to know them well, which I believe is good for them. But we lack knowledge, and there’s too little time to focus on mental health”. (Participant 13)

The analysis indicated that some of the participants were apprehensive when encountering patients with mental disorders and were intimidated by signs of mental ill-being. Although the participants felt secure in their relationship with patients, mental health seemed to be difficult to address, as one stated:

“We need more knowledge about mental disorders. I see that some of my colleagues are afraid or reluctant to visit patients with psychiatric diagnoses.” (Participant 7).

Lack of knowledge can lead to a fear of approaching patients with mental health disorders.

The participants’ statements suggested that the nurses were doing their best yet were afraid they might not be providing adequate care. Some expressed uncertainty about whether their actions for the patients were sufficient, and they were afraid of overlooking the bigger picture, as one explained:

“I feel confident in my knowledge and what I observe in my patients, but I’m unsure if I’m getting the full picture. The uncertainty about whether I know enough, if I’m asking the right questions, and if I should be doing more bothers me. That’s because I’m with the patient for such a short time, which means I risk missing the overall picture”. (Participant 6)

Such thoughts can lead to feelings of uncertainty and inadequacy among registered nurses that can result in a sense of moral distress, powerlessness and lower the quality of care. The participants generally felt that they had too little knowledge about signs of mental ill-being and that mental health was not prioritized in home care. They believed this was partly due to time constraints, giving them far too little time to spend with patients. They considered it important to work according to the law, to be professional, to maintain ethical awareness in patient interactions, and to see the whole person.

### Lack of opportunities to meet the patients’ needs regarding mental ill-being

The study found several barriers to focusing on signs of mental ill-being. Some participants mentioned that they understood the importance of focusing on mental health, but their everyday work pressure and limited time were barriers. Several felt that the municipality was avoiding responsibility. One participant said the following:

“I don’t have much time to talk about mental health when I’m with patients, so I end up not asking them about it. I just sort of do what I have to. When I think about it, it’s actually a way of avoiding responsibility. I learned about mental health in nursing school, and I know it’s important, so I should focus more on it”. (Participant 1)

The analysis revealed that it was often difficult to find the right words when discussing mental health and that many older people felt that mental health was still taboo. Several participants discussed the problem of mental health not being addressed. One stated:

“Mental health is not adequately addressed, and we don’t always realize when a patient is struggling with this because there are so many other issues surrounding elderly patients that take up more attention. The patients end up being bounced around in the system”.(Participant 3)

Several participants pointed to a lack of focus from management and expressed a desire for stronger engagement and encouragement from leadership, as one said:

“I wish there were a greater focus from management, such as organizing professional development days or allowing us to attend courses on mental health. That would help us gain more knowledge and keep the focus on mental health "alive." We have access to digital courses that we can complete individually, but there is never a time in our daily work to sit down and prioritize them. As a result, physical health ends up being prioritized instead”. (Participant 2)

The participants pointed out that municipal healthcare had difficulty with collaboration, which affected the patients. The participants were concerned that patients in need of mental health support were not receiving the help they required, partly due to poor collaboration between municipal services, as one mentioned. Additionally, there was little emphasis on mental health by home care management and organizational guidelines were lacking:

“Home care already has more than enough workload. We’re busy just making things work here and now. Unfortunately, there’s too little collaboration within the municipality. We should collaborate better and focus on how patients can receive the best help. Often, patients are referred to us because they also need physical healthcare, so home care is expected to help patients with everything, even if we don’t have the expertise for that”. (Participant 8)

Home nursing and mental healthcare thus seemed to find it difficult to collaborate on who should be responsible for caring for elderly patients with symptoms of loneliness and depression. The participants emphasized and aimed at person-centred responsibility for the mental health of elderly home-dwelling patients.

### Feelings of guilt for not doing enough for patients

The findings revealed that the participants experienced a range of emotions in their interactions with patients. Several talked about how their interaction with lonely and depressed elderly patients affected them, as one of them described:

“It’s when I sit down and think about how many older people are lonely and depressed that I also realize how little I know and how little experience I have. It’s frightening, and it affects the patients”. (Participant 11)

The participants became aware of their limited knowledge and how the patients suffered because of it. They felt that they had a significant lack of knowledge, but also recognized the need to create opportunities to acquire this knowledge.

The findings revealed great dedication to the patient group, with a deep desire to help them on a daily basis. The participants described feelings of fear and pain when talking about the patients they encountered in their daily work in home care. One explained it in this way:

“Many older people have told me that they’re not enjoying life in general and that they’d rather be dead. When they say this with such neutral indifference, it scares me. It feels painful”. (Participant 13)

The patients’ statements were agonizing for the participants to hear, leading to a sense of moral distress and powerlessness.

The study highlighted how signs of loneliness was manifested in the patients by a desire for the participants to stay with them as long as possible. Several participants explained how patients tried to keep the registered nurses with them in their home a little longer, noting that many of them had no other visitors:

“I often feel upset when I leave patients, with a gut feeling that I should have done more for them. They ask many questions, try to extend the visit and come up with other tasks to make the visit longer. They want me to stay longer. For some, the home care nurse might be the only visit they receive that day”. (Participant 12)

For some patients, home care is their only social contact as a participant explained:

“I think there are many older people who are lonely and appear downcast or depressed, even without having a formal diagnosis. Home care is often their only social contact. Some of them prepare coffee for my visit and hope that I will sit down and have a cup with them. It feels terrible to say that I don’t have time, and I can see the disappointment on their faces. It affects me deeply, and sometimes I carry the feeling of not being enough with me throughout the entire day”. (Participant 4).

The participants often found it painful to leave patients who needed more support than they could provide. They were deeply affected, and encountering lonely older people who were struggling and had few visitors made a significant impact on them. To describe their feelings and experiences, they used words like painful, distressing, difficult and tough, noting that these emotions often lingered long after they came home from work.

## Discussion

The aim of this study was to explore how registered nurses perceive their clinical competence in observing signs of loneliness and depression in older people in home care, and which barriers in healthcare services prevent quality improvement. The analysis of the interviews indicated that the participants generally felt confident in their interactions with patients. However, they also found a gap in their knowledge and skills related to observe signs of loneliness and depression, which they viewed as a significant professional hurdle. This could result in uncertainty and a feeling that they were not providing true person-centred care.

Furthermore, the participants noted that home care management only assumes a certain degree of responsibility for older people exhibiting signs of loneliness and depression. When their work conflicts with their professional principles, nurses may feel distressed or guilty for not providing enough support to their patients. Several situations led to moral distress, which occurred when the participants knew the ethically correct action to take but instead felt powerless to take action. The study identified several factors influencing the participants’ ability to recognize signs of loneliness and depression in older home-dwelling people. It also revealed several obstacles that hindered the participants from enhancing their clinical skills and gaining practical experience.

### Knowledge builds confidence in encounters with patients

The results of this study revealed that all the participants lacked knowledge about loneliness and depression. This knowledge gap is likely to negatively affect patients, as they may not receive appropriate preventative or therapeutic interventions. This concurs with research highlighting that home care nurses often feel uncertain about how to manage patients with signs of loneliness and depression due to a lack of knowledge [53]. Further, it does not align with a person-centred approach, which ensures that services are designed around the needs and preferences of the individuals who use them, rather than focusing on the convenience of the providers [27]. As a result, patients may not receive necessary nursing interventions in terms of health promotion, prevention, recovery or rehabilitative care [54]. The importance of knowledge is highlighted in the study by Karlsson et al. [55], which points out that nurses play a key role in identifying signs of mental ill-being in patients. That study further emphasizes that, alongside knowledge, factors such as time, collaboration with other professionals, and cooperation with family members are essential for establishing best practices [55]. A study reveals the essential nature of home care nurses’ role and clinical competence, as they are close to patients in their own homes, allowing them to build meaningful, trusting person-centred relationships by having open communication with patients [56]. When patients can make decisions about their care and treatment in partnership with nurses, they may be more satisfied with their care, which is also an important aspect of the person-centred nursing approach [27]. Therefore, it is crucial that home care nurses are qualified to identify signs of mental ill-being such as loneliness and depression, ensuring that care is tailored to the individual needs and preferences of each patient, as also shown by Waidman et al. [54]. Person-centred care also benefits nurses, as increased patient engagement enhances staff performance and morale [27]. This can motivate registered nurses to improve their knowledge and skills in mental health, raise awareness of ethical values in nursing practice, boost nurses’ confidence in patient interactions, and ultimately improve the quality of healthcare in accordance with ethical guidelines and legislation [33, 43]. Kwame and Petrucka [57] argue that interaction between registered nurses and patients requires effective communication to ensure person-centred care, while knowledge is essential for communicating effectively. This is particularly important because many patients may have signs of loneliness and depression that are not always obvious, and without proper training, such symptoms can be overlooked or misunderstood, as pointed out in previous research [9, 15, 18]. Moreover, delivering healthcare services that respect and meet patients’ needs is essential for high-quality care, and effective communication between patients and nurses is key to achieving patient-centred home care [58].

Research highlights that the interaction between registered nurses and patients is a vital resource for enhancing the quality of life among older people. Establishing strong nurse-patient relationships fosters a sense of care and trust, enabling nurses to identify and assess signs of loneliness and depression by listening to patients share their experiences. This process requires both time and expertise in mental health. While home care nurses possess the skills to observe indicators of loneliness and depression, these observations may go unaddressed if they are not integrated into the documentation system. Consequently, signs of moral distress may remain undetected, leading to an incomplete assessment of the patient's mental health, as noted in previous studies. The ability to document effectively, along with the use of assessment tools designed to ensure thorough and consistent documentation, is essential for conducting a comprehensive evaluation of patients' needs [21, 59, 60].

### Professional responsibilities

Quality in healthcare is closely linked to person-centred care [27]. To improve health and healthcare outcomes, healthcare systems must find effective ways to implement a person-centred approach [61]. Furthermore, the present study indicates a possible gap between the level of competence nurses in municipal healthcare are expected to have and the competence that actually exists. However, this research also indicates a general lack of opportunities for skills development in municipal healthcare. This represents a notable gap, suggesting that the municipality could take more responsibility in addressing the need for training to enhance the quality of mental healthcare in home care nursing, which is also in line with previous research [62]. It is also the management’s duty to ensure that staff have sufficient competence, and to encourage and provide opportunities for training in line with the Working Environment Act [41]. The Health Personnel Act points out that registered nurses have a personal responsibility to improve their nursing skills to gain confidence in their role [43]. This involves not only the individual registered nurse’s values and attitudes but also the ethical perspective of nursing practice. The professional ethical guidelines for nurses emphasize that each registered nurse is responsible for a practice that promotes health and prevents illness and suffering [38, 43]. It is thus essential for home care nurses to provide coordinated care and offer personalized support or treatment for their patients in line with the person-centred care approach [27]. The Norwegian government has consistently placed a high priority on enhancing mental healthcare and has stressed the importance of providing support and care for older people in their own homes [63]. In addition, the Health Personnel Act states that municipal healthcare workers are responsible for delivering patient-centred and preventative care, treatment and support to patients with both physical and mental health problems [38]. Improved collaboration between home care services and mental healthcare services is needed for patients to receive vital healthcare services. Failing to focus on person-centred care can also be considered as neglecting one’s duty, with a high risk of missing important signs and conditions [27] and might lead to inadequate municipal healthcare services.

The participants in this study underlined several barriers that prevented them from focusing on mental health, such as limited time, high workload and a focus on physical healthcare.

Municipal healthcare management seems to prioritize mental health at a lower level, which might negatively impact patients. This contradicts the legislation, which states that municipalities must promote health, prevent illness, and provide care and support. When a patient relies solely on home nursing, mental health should be given the same priority as physical health in home care [33]. Nevertheless, it is crucial to recognize that the participants involved in this study probably performed to the best of their abilities while working independently. Furthermore, the importance of preventative work is paramount and is stipulated in the Health and Care Services Act [33]. At the same time, nurses must adhere to the home care regulations regarding assessments and tasks. This might create a gap between the needs of patients and the focus of home care services. The barriers in this study made it difficult to provide comprehensive care, which is a critical issue in line with quality in home care. According to Djukanović et al. [64], recognizing signs of loneliness and depression in older people and providing appropriate care is vital to improve their quality of life [64].

### Moral distress in home care

This study revealed that many of the participants were emotionally affected not only by the large number of older people struggling with signs of loneliness and depression but also by how seldom they received relevant nursing interventions. This leads to moral distress among the home care nurses because of the gap between the function of the service system and person-centred care [65]. They realized that municipal healthcare had few interventions they could implement for older people with sign of loneliness and depression, which upset the nurses. To witness this situation without being able to help these people was difficult for the participants, who felt that they constantly had to compromise on what they could offer the patients. Despite time constraints, the registered nurses attempted to give the patients “a little extra” by postponing other tasks; they tried to spend more time with the patients and listen to what they had to say. This is in line with other research that points out that registered nurses may stay with patients longer than their mandate, chatting about everyday topics or providing supportive discussions. This places nurses in a dilemma between meeting patient needs and fulfilling the requirements of the organization [16]. Registered nurses aim to be present for their patients, demonstrating compassion and respect, and understanding what is important to the individual, which is also in line with person-centred care [27]. Halvorsrud et al. [16] highlight the lack of continuity in interventions. The lack of opportunity for continuity deprives home care nurses of the professional freedom to provide follow-up care over time [16].

The findings revealed that the registered nurses were doing their best and strove to provide person-centred care in line with Dostálová et al. [66], but felt that it was not enough. This led to moral distress, guilt and a sense of inadequacy. Many of the registered nurses reported that patients with signs of loneliness and depression often fell between the cracks, receiving inadequate care due to an unclear division of responsibility within healthcare and the low priority of mental health in home care. The Norwegian authorities [63] have long claimed to focus on and prioritize mental health, but in home care at least, this study seems to suggest otherwise. According to Tomstad et al. [23], some older people try to prolong conversations with their nurses by asking new questions as the nurses prepare to leave, sometimes inviting them to stay for coffee, as also was revealed in this study. The study shows how deeply affected the participants were, and many described it as an emotionally painful experience leading to moral distress and powerlessness among the registered nurses, who continually compromise on their values and beliefs, as seen in the study by Bentzen et al. [67]. This research argue that registered nurses recognize that ethical values are essential for delivering high-quality care, yet these values are frequently sidelined in everyday practice. This leads to feelings of frustration, burnout and guilt among registered nurses. To address this issue, there is a pressing need for systemic changes that can foster a caring culture benefitting both patients and registered nurses [67]. This presents an ethical dilemma that registered nurses are required to face daily [43].

Even though addressing mental health issues was challenging and often remained an under-communicated topic for many of the participants, this study revealed that the registered nurses still perceived their role and work as a valuable opportunity for building relationships with their patients. They found that home nursing was an excellent setting to get to know patients well, create a safe and trusting relationship, and be in a unique position to adopt a person-centred care approach [27]. In a person-centered approach, it is essential for home care nurses to be attentive to the needs of those seeking care and to build trust [58]. Care is experienced through the encounter between the registered nurse and the patient. For a caring relationship to be truly caring, nurses must understand the patient’s subjective experience of the relationship. By offering care, the registered nurse has a unique opportunity to establish a meaningful relationship with the patient, adopt a person-centred approach and prevent moral distress. One way to achieve person-centred care is to show respect for patients, have compassion for the work that needs to be done, and always focus on dignity when interacting with patients [27].

## Strengths and limitations

To ensure credibility and transferability, the authors aimed to provide a comprehensive description of the study and included quotes that accurately reflected the participants’ perspectives. Dependability was addressed by adhering to the analytical steps outlined by Graneheim and Lundman [47]. The research team engaged in discussions about their preconceptions to ensure that they did not influence the interviews. The entire team participated in the analysis process and contributed to developing all parts of the article [46, 47]. Reflexivity was a continuous topic of discussion throughout the research process, with careful consideration given to preconceptions, personal biases, and the theoretical frameworks employed, in order to enhance credibility. However, the authors’ preconceptions and biases may have impacted the final manuscript [44].

Telephone interviews offer an efficient and accessible method of collecting data over a wide geographical area, while also safeguarding participant anonymity [68]. The researchers contacted participants from urban and rural areas throughout Norway. However, the lack of non-verbal cues in telephone interviews might have limited the possibility to gather certain critical information. Although the telephone interviews provided a wealth of data, face-to-face interactions might have yielded further insights in these interviews.

Conducting the research in Norway was valuable for highlighting the need for home nursing to consider signs of loneliness and depression issues in older people, and because few such studies have been conducted on municipal healthcare services. A strength of the study is that fifteen out of thirty-five municipalities agreed to participate, making a varied sample. Although participants from all regions of Norway were included, only a small number of municipalities were represented. Nevertheless, it was a significant advantage to have participants from different municipalities in order to provide an impression of variations in municipal healthcare in Norway.

This study in the Norwegian context may offer transferable insights to countries with comparable healthcare systems.

The authors of the study have limited clinical experience working in home care. It could be argued that this made it easier to ask questions to the participants to gather valuable information. Efforts were made to set aside preconceptions so that the authors would not influence the participants or the content of the text through interpretations [44].

## Discussion of the ethical issues

The study was aimed to respect autonomy by acknowledging and respecting each participant's right to make their own choices and decisions. Autonomy refers to their ability and right to decide for themselves, based on their own values, desires, and judgments. To respect autonomy, the authors ensured that both verbal and written information was provided, allowing participants to decide whether to participate in the study and to make an informed decision about their involvement. The principle of non-maleficence, which entails avoiding actions that could cause physical, psychological, or social harm, was also prioritized. This involves always assessing whether the potential benefits of an action outweigh the possible risks. In such a study, participants may provide very important information that could contribute to the quality improvement of practice, suggesting that the benefits outweigh the harm. The non-maleficence principle requires that potential consequences are carefully considered and that the risk of harm is minimized [69].

The study was performed in accordance with the Declaration of Helsinki [51]. The interviews were conducted based on informed consent, voluntary participation, and the participants' desire to contribute to the study. Thus, a utilitarian perspective has been maintained, where the right action is the one that produces the greatest benefit or happiness for the most people (overall well-being for all involved). In this context, this applies to older home-dwelling people who show signs of mental ill-being [70].

The participants' confidentiality was ensured by anonymizing the data, using codes instead of names, and storing interview data in a secure location (TSD). Any personal details that could identify the participants remained confidential. The participants' potential stress was managed by creating a safe and supportive interview environment, where they were informed that they could stop or withdraw from the study at any time without any consequences. It was sought to ensure that the participants felt heard [44].

## Suggestions for further research, and recommendations for practice and education

There is a need for further research on understanding registered nurses’ feeling of uncertainty in clinical practice to develop and implement strategies that effectively support them [71]. There is also a need to raise awareness of signs of mental ill-being in older people [72], and additional research is essential to enhance knowledge and understanding of registered nurses’ perceived competence in identifying signs of loneliness and depression in elderly home care patients to detect gaps and improve clinical practice [16, 73]. It should also be researched whether the quality of nursing education has any correlation with levels of loneliness. To improve clinical practice, it is essential to enhance registered nurses’ knowledge of mental health by providing targeted training that focuses on recognizing signs of loneliness and depression in older people. Such training should also equip registered nurses with strategies for asking meaningful questions and addressing patients’ responses effectively. Furthermore, clinical practice should emphasize the importance of relationship-based care by allowing registered nurses sufficient time and opportunities to build trust and meaningful connections with their patients. This approach can help nurses better understand the emotional and psychological needs of older people. Additionally, fostering collaboration between home care services and mental health professionals is crucial to ensure that older people receive comprehensive and coordinated support. From an educational perspective, integrating practical training methods, such as case studies and role-playing exercises can be meaningful, to prepare registered nurses to address mental health issues in real-world scenarios. Reflective exercises and peer support groups incorporated into the educational programs is also a way of helping registered nurses to process the emotional challenges they may face when working with older people with signs of mental ill-being. Finally, ethics and prioritization should form a core part of the curriculum in nursing education, enabling nurses to navigate ethical dilemmas and to prioritize mental health as an integral component of care for older home-dwelling patients.

## Conclusions

Lack of education and training in mental health creates uncertainty and limits registered nurses’ ability to effectively address signs of loneliness and depression. Registered nurses often feel unprepared to handle psychological aspects of care.

Organizational barriers hinder effective care. Additionally, the emotional toll on registered nurses, who experience guilt and moral distress due to their limited ability to provide sufficient care, highlights the impact of these systemic issues on patients.

The study underscores the need for improved training, better collaboration, and stronger leadership to prioritize mental health and ensure that nurses have the necessary resources and support to meet the needs of older people with signs of mental ill-being in home care.

## Supplementary Information

Supplementary Material 1.

## Data Availability

All data (generated or) analysed during this study are included in this published article [and its supplementary information files].
