# Supplementary material for: Nurses’ perspectives on clinical competence to identify loneliness and depression in older people in home care: a qualitative study
Source: BMC Health Serv Res. 2025 Mar 12;25:370. doi: 10.1186/s12913-025-12428-y (PMC11905625; doi:10.1186/s12913-025-12428-y)
Supplement: Supplementary file 1 — Supplementary Material 1. [file 12913_2025_12428_MOESM1_ESM.docx]

Interview guide

1. Please tell me about your experience of observing loneliness and depression in patients. What do you observe, and how do you observe it?

- Positive experiences?
- Negative experiences?

1. Describe your experience of potential barriers of observing loneliness and depression.

- What do you think about this?

1. What feelings do you have related to working in home healthcare? How does work in home healthcare influence your work as a professional nurse?

- Positive or/and negative experiences?
